# Curcumin-Induced Heme Oxygenase-1 Expression Prevents H_2_O_2_-Induced Cell Death in Wild Type and Heme Oxygenase-2 Knockout Adipose-Derived Mesenchymal Stem Cells

**DOI:** 10.3390/ijms151017974

**Published:** 2014-10-08

**Authors:** Niels A. J. Cremers, Ditte M. S. Lundvig, Stephanie C. M. van Dalen, Rik F. Schelbergen, Peter L. E. M. van Lent, Walter A. Szarek, Raymond F. Regan, Carine E. Carels, Frank A. D. T. G. Wagener

**Affiliations:** 1 Department of Orthodontics and Craniofacial Biology, Radboud University Medical Center, Radboud Institute for Molecular Life Sciences, PO Box 9101, 6500 HB Nijmegen, The Netherlands; E-Mails: niels.cremers@radboudumc.nl (N.A.J.C.); ditte.lundvig@radboudumc.nl (D.M.S.L.); carine.carels@radboudumc.nl (C.E.C.); 2 Department of Rheumatology, Experimental Rheumatology, Radboud University Medical Center, Radboud Institute for Molecular Life Sciences, PO Box 9101, 6500 HB Nijmegen, The Netherlands; E-Mails: stephanie.vandalen@radboudumc.nl (S.C.M.D.); rik.schelbergen@radboudumc.nl (R.F.S.); peter.vanlent@radboudumc.nl (P.L.E.M.L.); 3 Department of Chemistry, Queen’s University, Kingston, ON K7L 3N6, Canada; E-Mail: szarekw@chem.queensu.ca; 4 Department of Emergency Medicine, Thomas Jefferson University, Philadelphia, PA 19107, USA; E-Mail: Raymond.regan@jefferson.edu

**Keywords:** adipose-derived mesenchymal stem cells, oxidative stress, apoptosis, heme oxygenase, carbon monoxide

## Abstract

Mesenchymal stem cell (MSC) administration is a promising adjuvant therapy to treat tissue injury. However, MSC survival after administration is often hampered by oxidative stress at the site of injury. Heme oxygenase (HO) generates the cytoprotective effector molecules biliverdin/bilirubin, carbon monoxide (CO) and iron/ferritin by breaking down heme. Since HO-activity mediates anti-apoptotic, anti-inflammatory, and anti-oxidative effects, we hypothesized that modulation of the HO-system affects MSC survival. Adipose-derived MSCs (ASCs) from wild type (WT) and HO-2 knockout (KO) mice were isolated and characterized with respect to ASC marker expression. In order to analyze potential modulatory effects of the HO-system on ASC survival, WT and HO-2 KO ASCs were pre-treated with HO-activity modulators, or downstream effector molecules biliverdin, bilirubin, and CO before co-exposure of ASCs to a toxic dose of H_2_O_2_. Surprisingly, sensitivity to H_2_O_2_-mediated cell death was similar in WT and HO-2 KO ASCs. However, pre-induction of HO-1 expression using curcumin increased ASC survival after H_2_O_2_ exposure in both WT and HO-2 KO ASCs. Simultaneous inhibition of HO-activity resulted in loss of curcumin-mediated protection. Co-treatment with glutathione precursor *N*-Acetylcysteine promoted ASC survival. However, co-incubation with HO-effector molecules bilirubin and biliverdin did not rescue from H_2_O_2_-mediated cell death, whereas co-exposure to CO-releasing molecules-2 (CORM-2) significantly increased cell survival, independently from HO-2 expression. Summarizing, our results show that curcumin protects via an HO-1 dependent mechanism against H_2_O_2_-mediated apoptosis, and likely through the generation of CO. HO-1 pre-induction or administration of CORMs may thus form an attractive strategy to improve MSC therapy.

## 1. Introduction

Administration of mesenchymal stem cells (MSCs) forms a promising novel adjuvant treatment to improve tissue repair [1,2,3,4]. MSCs have been shown to accelerate dermal wound healing and can regenerate diverse injured organs, such as kidney, lung and heart [5,6,7,8,9]. MSCs are a heterogeneous population of fibroblast-like, multipotent stem cells characterized by their ability to differentiate into mature cells of the mesodermal lineage, like osteoblasts, chondrocytes, adipocytes, endothelial cells, and myocytes, and into cells outside the mesodermal lineage, such as keratinocytes, and fibroblasts [3,10,11,12]. Adipose tissue forms an easy accessible source for isolating MSCs for the use in (autologous) regenerative medicine and may therefore be a better alternative than MSCs isolated from other sources, such as bone marrow (BM) [13,14,15,16]. These adipose-derived MSCs (ASCs) are abundantly present in adipose tissue, easy to isolate, and highly proliferative. In addition, they secrete extensively regenerative factors, such as hepatocyte growth factor, vascular endothelial growth factor, basic fibroblast growth factor, interleukin (IL)-6, -7, -8, and -11, and stromal cell-derived factor-1 (SDF-1) [14,15,16].

Despite the promising utilization of MSCs in regenerative medicine, the low survival after administration and the limited migration of MSCs to the site of injury limits their therapeutic efficacy [17,18]. Local administration to the site of injury may attenuate the problems with MSC migration. However, MSC administration in the vicinity of the injured tissue may also affect MSC survival, as the wound microenvironment harbors excessive levels of inflammatory and oxidative mediators, hypoxia, and limited blood flow [19]. Oxidative and inflammatory stresses are known causes of MSC death [17,18,20]. Cells have several mechanisms to protect themselves against inflammatory and oxidative insults, including cytoprotective enzyme systems (e.g., glutathione *S*-transferase, dismutases, catalases, and peroxidases) or anti-oxidants (vitamin A, C, and E, urate, glutathione, and bilirubin) [21]. In addition, MSCs may upregulate anti-apoptotic and anti-oxidative genes [16,19]. Pre-induction of cytoprotective pathways in the ASCs may improve their therapeutic potential by protecting them against the harsh microenvironment [22,23].

The cytoprotective enzyme heme oxygenase (HO) is important for (stem) cell survival and functioning [17,18,24,25]. HO breaks down heme into biliverdin, free iron (Fe^2+^) and carbon monoxide (CO). Biliverdin (BV) is rapidly converted into bilirubin (BR) by biliverdin reductase [26,27]. The iron scavenger ferritin is co-induced by HO-derived iron and is important for protection against iron-mediated reactive oxygen species (ROS) formation [28]. Two distinct isoforms (HO-1 and HO-2) exist. HO-2 is mainly constitutively expressed whereas HO-1 is highly inducible by a variety of patho-physiological stimuli, such as free heme, cytokines, hypoxia, and oxidative stress [29]. HO-2 is responsible for maintaining normal metabolic cellular functions, and regulating physiological levels of ROS [30,31,32].

Induction of HO-1 has been demonstrated to improve MSC therapy *in vivo* by improving tissue functioning of the damaged organ, whereas inhibition of HO-activity worsened MSC therapy outcome in diverse pathologic conditions, such as ischemia reperfusion injury of the heart, pulmonary arterial hypertension, diabetes, and dermal wound healing [31,33,34,35,36,37,38,39,40]. It has been shown *in vitro* that HO-1 overexpression increases BM-derived MSC survival against oxidative stress, but the exact mechanism remains unknown [17,34]. By contrast, induced pluripotent stem cells (iPSCs) and murine embryonic fibroblasts (MEFs) derived from HO-1 knockout mice accumulate higher levels of intracellular ROS after exposure to oxidative stress [41,42]. Moreover, HO-1 KO iPSCs and MEFs are more sensitive to hydrogen peroxide (H_2_O_2_)-induced cell death [41,42]. Since, the effects of HO-1 are extensively studied in stem cells, we investigate here the role of the HO-2 isoform in protection against oxidative stress. HO-2 has been shown to act in a protective manner against inflammatory and oxidative injury and cell apoptosis in diverse cell types [43,44,45,46,47,48,49,50,51].

In the present study we postulated that HO-2 WT ASCs would be better protected against oxidative stress when compared to HO-2 deficient ASCs. Additionally, we aimed to investigate the role of HO-activity and HO-effector molecules BR/BV and CO on ASC survival after H_2_O_2_-induced oxidative stress.

## 2. Results and Discussion

### 2.1. Isolation and Characterization of Adipose-Derived MSCs (Mesenchymal Stem Cells) (ASCs)

**Figure 1:**
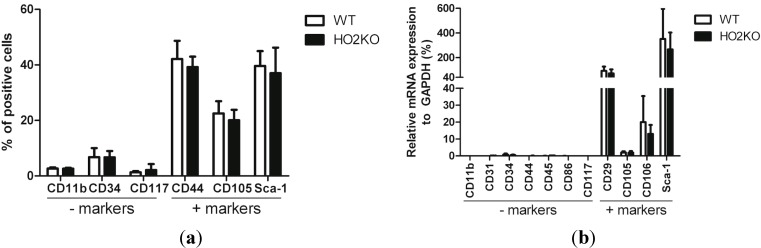
Phenotypic analysis of isolated WT (wild type) and HO-2 KO ASCs. (**a**) Percentage of positive cells for ASC exclusion markers (negative) and ASC cell surface markers (positive) of isolated WT and HO-2 KO ASCs, measured using flow cytometry; (**b**) mRNA levels of negative and positive ASC markers of isolated WT and HO-2 KO ASCs, analyzed by qPCR and presented as 2^−Δ*C*t^. No significant differences were observed between in the expression of selected markers between WT and HO-2 KO ASCs using both flow cytometry and qPCR. Data represent mean ± SD of three independent experiments.

ASCs were isolated from mouse (WT and HO-2 KO) adipose tissue around the inguinal lymph nodes and cultured in defined media as described here in Experimental Section 3. The cells adhered to plastic and had, as expected for ASCs, a fibroblast-like morphology. Passage three of the isolated cell populations were characterized for the expression of mesenchymal, endothelial, and hematopoietic markers by immunophenotyping (Figure 1a) and qPCR (Figure 1b). Surface antigen expression of the isolated cells, using flow cytometry, was consistent with literature data and our previous results [52]. The cells expressed ASC surface markers Sca-1, CD44, and CD105 and were negative for the exclusion markers CD117 (hematopoietic stem cells), CD11b (macrophages) (Figure 1a), and showed low expression of CD34 (endothelial/hematopoietic progenitor cells) [16,53,54,55,56,57,58]. No significant differences in ASC marker expression was detected between WT and HO-2 KO ASCs. These phenotypic observations at the protein level were further corroborated at the mRNA level using common MSC markers (Figure 1b). The isolated cells expressed high mRNA levels of ASC markers Sca-1, CD29 [16,54,55,56,58], CD105, and CD106 [16,54], whereas cells were negative for exclusion markers CD11b, CD31 [16,53,54,55,56,57], CD34, hematopoietic marker CD45 [16,53,54,55,56,57,58], antigen-presenting cell marker CD86 [58], and CD117 showing low levels of mRNA expression, compared to housekeeping gene GAPDH. No significant difference in marker expression was found between WT and HO-2 KO ASCs. We conclude that the isolated cells are ASCs, since the marker expression profile corresponded well with literature data of earlier reported isolated murine ASCs.

### 2.2. H_2_O_2_ Induces Cell Death in ASCs in a Dose-Dependent Manner Independently from HO-2 Expression

ASCs were exposed to increasing concentrations of H_2_O_2_ (0–500 µM) to investigate the effect of oxidative stress on ASC survival. H_2_O_2_ is a strong oxidant that can cause cell death and apoptosis. Compared to other ROS, H_2_O_2_ is a relatively long-lived molecule commonly used in models of oxidative stress [59]. Exposing cells to H_2_O_2_ results in deleterious effects of hydroxyl and peroxyl radicals on membrane lipids and proteins, resulting in loss of mitochondrial membrane potential, mitochondrial dysfunction, and eventually apoptosis [60,61].

ASCs were exposed for 24 h to increasing concentrations of H_2_O_2_ and cell viability was assessed using the Alamar blue assay. This assay is based on the ability of metabolically active cells to convert the Alamar blue reagent into a fluorescent signal, which is directly proportional to the cellular viability. Damaged and non-viable cells have lower innate metabolic activity, and generate thus a proportionally lower signal.

**Figure 2:**
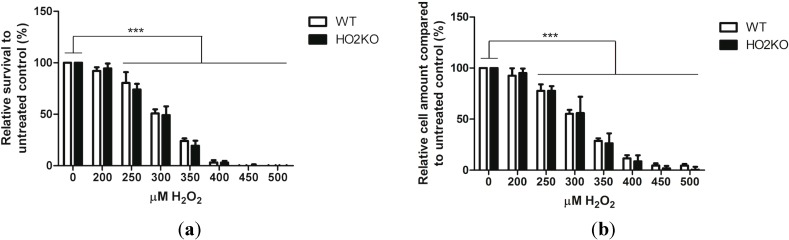
Survival and cell amount of isolated WT and HO-2 KO ASCs after exposure to increasing doses of H_2_O_2_ (0–500 µM) for 24 h. (**a**) WT and HO-2 KO ASC survival and (**b**) amount of ASCs after H_2_O_2_-treatment were analyzed using the Alamar blue assay and picogreen assay, respectively. Doses of 250 µM H_2_O_2_ and higher resulted in a significant decreased survival and, subsequently, lower cell amounts (all *p* < 0.001) compared to untreated control. No significant differences were observed between WT and HO-2 KO ASCs at any concentration. A dose of 350 µM H_2_O_2_ was chosen in further experiments to induce ASC death. Data represent mean ± SD of three independent experiments with four samples per condition. * is significant different from treated control. (*** *p* < 0.001).

ASC viability decreased gradually after exposure to increasing concentrations of 200–500 µM H_2_O_2_ for 24 h (Figure 2a). Exposure to doses of 250 µM H_2_O_2_ and higher had a significant cytotoxic effect for both WT and HO-2 KO ASCs compared to controls (all *p* < 0.001). No ASCs survived at concentrations of 450 µM H_2_O_2_ and higher. In parallel, the amount of cells after 24 h treatment with H_2_O_2_ were analyzed using the picogreen assay. This assay quantifies the amount of double stranded DNA and is directly proportional to the amount of cells. These results were similar and corroborated our findings with the Alamar blue assay. The amount of cells decreased significantly at H_2_O_2_ concentrations of 250 µM and higher, in a dose-dependent fashion for both WT and HO-2 KO ASCs (Figure 2b). After treatment with 350 µM H_2_O_2_ 24% ± 2.5% of WT and 19.5% ± 4.9% of HO-2 KO ASCs survived. At this concentration, we found increased apoptosis in WT and HO-2 KO ASCs as measured by the increased levels of phosphatidylserine flip-flop and nuclear staining, using Annexin V-FITC and propodium iodide (PI), respectively (see Figure S1). Surprisingly, no significant difference could be observed between WT and HO-2 KO ASCs in sensitivity towards H_2_O_2_-induced cell death at any measured concentration. This suggests that HO-2 does not protect against H_2_O_2_-induced ASC death. Probably, the HO-2-activity level in naïve ASCs is too low. Although HO-2 has been demonstrated to protect against a wide range of oxidative insults in diverse cells, the role of HO-2 may be cell and stressor dependent [43,44,46,49]. HO-2 can either protect or augment apoptosis [45,50]. In this setting, ASCs were not dependent on HO-2 for their protection against H_2_O_2_-induced cell death.

Based on the above data, 350 µM H_2_O_2_ was chosen as cytotoxic dose since it offers an analytical window to assess whether the HO-system could mediate protection against oxidative stress-induced cell death in ASCs.

### 2.3. Can Curcumin Induce HO-1 Expression in ASCs?

**Figure 3:**
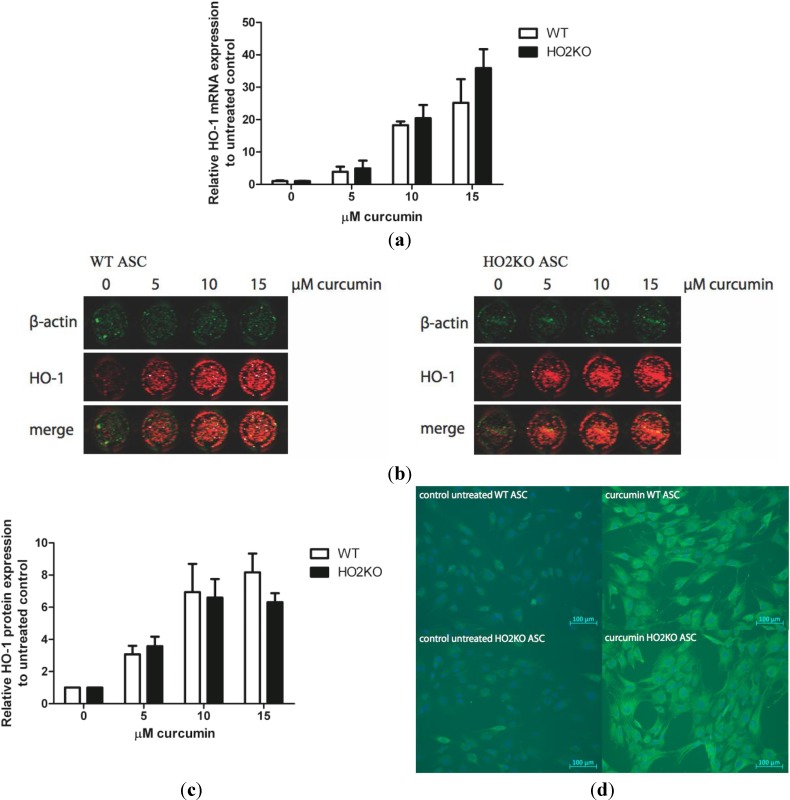
HO-1 mRNA and protein expression was induced with increasing doses of curcumin (0–25 µM) for 24 h in isolated WT and HO-2 KO ASCs. (**a**) WT and HO-2 KO ASCs were treated with curcumin for 24 h and HO-1 mRNA expression was evaluated with qPCR, corrected for the housekeeping gene *GAPDH* and normalized to untreated control. HO-1 mRNA expression was significantly (*p* < 0.05) induced at every concentration in comparison to untreated control. Data represent mean ± SD of three independent experiments. No significant differences between WT and HO-2 KO ASCs were found; (**b**) WT and HO-2 KO ASCs were treated with curcumin for 24 h and HO-1 protein expression was evaluated with In-Cell Western (green: β-actin, and red: HO-1); (**c**) Quantification of the In-Cell Western signal, corrected for the amount of cells with β-actin and related to untreated control. HO-1 protein expression was significantly (*p* < 0.05) induced at every concentration in comparison to untreated control. Data represent mean ± SD of three independent experiments. Four samples were analyzed per condition. No significant differences between WT and HO-2 KO ASCs were found; (**d**) WT and HO-2 KO ASCs were untreated or treated with 10 µM curcumin for 24 h and HO-1 protein expression was evaluated using immuno-fluorescent staining of chamberslides (green is HO-1, blue represents nuclear staining with DAPI). Data is representative of three independent experiments.

Next, we studied if we could induce the expression of HO-1 in ASCs to provide protection against oxidative damage. Therefore, ASCs were treated with increasing curcumin concentrations for 24 h and *HO-1* gene transcription was subsequently assessed by qPCR (Figure 3a).

Exposure to curcumin for 24 h induced HO-1 mRNA levels dose-dependently in WT and HO-2 KO ASCs compared to untreated control. No significant differences in HO-1 induction were found between the two genotypes.

Next, the HO-1 protein expression after treatment with different concentrations of curcumin was assessed using In-Cell Western (Figure 3b). HO-1 protein expression was quantified in relation to β-actin protein expression (Figure 3c). We observed that HO-1 protein expression was significantly and dose-dependently induced after 24 h treatment with increasing doses of curcumin compared to untreated control in both WT and HO-2 KO ASCs. HO-1 protein expression after induction with curcumin was not significantly different between WT and HO-2 KO ASCs at any of the used concentrations. Furthermore, a plateau in expression was reached as we did not detect any difference in HO-1 expression levels at concentrations of 10 and 15 µM. For our next experiments we used 10 µM curcumin, since high concentrations of curcumin (25 µM) may cause cell death *in vitro* [62]. Treatment with 10 µM curcumin did not result in cytotoxic effects and induced HO-1 mRNA expression 20-fold and HO-1 protein levels roughly 7-fold compared to untreated cells.

The HO-1 protein induction in WT and HO-2 KO ASCs was also measured using immuno-fluorescent staining (Figure 3d). In accordance with the mRNA and In-Cell Western expression data we demonstrated that HO-1 protein expression was highly up-regulated in ASCs from both genotypes after treatment with 10 µM curcumin in comparison with untreated control.

There were no significant differences observed in HO-1 induction at either mRNA or protein level between WT and HO-2 KO ASCs after treatment with 10 µM curcumin. We demonstrated that curcumin acts as a potent inducer of HO-1 in both WT and HO-2 KO ASCs after treatment for 24 h.

### 2.4. HO-1 Inducer Curcumin Protects against H_2_O_2_-Mediated ASC Death in a HO-2 Independent Manner

Pre-conditioning of MSCs with cytoprotective factors can improve MSC survival against several injurious stressors *in vitro*, such as serum-free conditions, hypoxia and oxidative stress [14,63]. It has been shown that pre-conditioning of MSCs with melatonin [61], NO-donor *S*-nitroso *N*-acetylpenicillamine (SNAP) [64], or overexpression of transcription factor nuclear factor-erythroid 2-related factor 2 (nrf2) [18] can prevent MSC death caused by H_2_O_2_. Interestingly, these preconditioning factors are also potent inducers of HO-1 [65,66,67]. Since HO-1 can protect against oxidative stress in diverse models [17,34], we investigated if HO-1 induction can also protect against oxidative stress-induced ASC death. Since HO-1 transfection is difficult to introduce safely into the clinic, we investigated the use of the safe pharmaceutical HO-1 inducer curcumin. Curcumin is a natural product that is already used in daily life as a spice in the Indian kitchen [68,69], acts as an anti-oxidant [70], and provides protection against a wide variety of diseases and conditions [71,72,73]. Moreover, a protective role for polyphenols, including curcumin, on BM-derived MSC survival against oxidative stress was recently demonstrated [21]. However, any relation to the HO-system was not investigated, and the survival was thought to relate to the induction of ROS-reducing enzymes glutathione peroxidase (GPX) and catalase [21].

**Figure 4:**
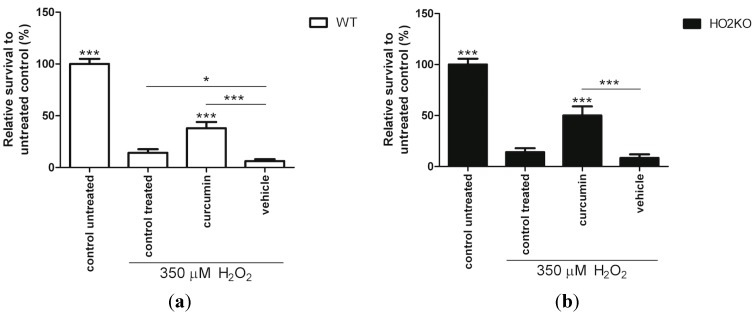
Curcumin treatment rescues WT and HO-2 KO ASCs against H_2_O_2_-induced cell death. (**a**) WT ASC and (**b**) HO-2 KO ASC survival after 24 h pre-treatment with curcumin, vehicle control, or untreated (control) and 24 h co-treatment together with 350 µM H_2_O_2_following an Alamar blue assay and fluorimetric quantification, related to untreated control. Vehicle consisted of 0.5% ethanol (curcumin solvent) in culture media. * is significant different from H_2_O_2_-treated control. (* *p* < 0.05, *** *p* < 0.001). Data is representative of three independent experiments and presents mean ± SD. For each condition six samples were analyzed.

In order to investigate the role of curcumin on H_2_O_2_-induced cell death in more detail, ASCs were pre-treated for 24 h with 10 µM of curcumin followed by co-treatment with 350 µM H_2_O_2_ for 24 h after which the cell viability was assessed using the Alamar blue assay. Pre-conditioning with curcumin significantly improved ASC survival during oxidative stress (Figure 4). Pre-treatment with 10 µM curcumin increased ASC survival 2.7- and 3.6-fold in WT (Figure 4a) and HO-2 KO ASCs (Figure 4b), respectively, compared to cells exposed to solely 350 µM H_2_O_2_. This is further supported by picogreen data that showed significantly more WT and HO-2 KO ASCs when pre-treated with curcumin (Figure S2). Vehicle control ethanol did not increase ASC survival and cell amount compared to H_2_O_2_-treated control cells. Since we found that curcumin acts as a strong inducer of HO-1 this suggests a possible role for HO-1 in ASC survival from oxidative stress.

Interestingly, transient overexpression of the human *HO-1* gene in BM-derived MSCs has been shown to protect against oxidative stress-induced cell death [17,34]. HO-1 transfected BM-derived MSCs were more resistant to cell death than non-transfected MSCs after exposure to H_2_O_2_ [34]. Survival at 500 µM H_2_O_2_ for two hours was higher after HO-1 transfection and HO-1 induction could thus protect BM-MSCs to a short exposure of high concentrations H_2_O_2_. At a lower concentration of H_2_O_2_ but longer treatment time with H_2_O_2_ we showed also protection for ASC following curcumin exposure, suggesting that HO-1 induction plays a role. We further investigated the downstream mechanism of protection after pre-conditioning with the clinically more relevant curcumin towards an exposure of H_2_O_2_ for 24 h.

In summary, curcumin provides similar protection against H_2_O_2_-induced cell death both in WT and HO-2 KO ASCs. A possible involvement of HO-activity will be further evaluated.

### 2.5. Is Rescue from H_2_O_2_-Induced ASC Death by Curcumin Mediated by HO-Activity?

We have shown that curcumin can protect against ASC death caused by oxidative stress. Since we demonstrated that curcumin induces HO-1 expression in ASCs, we next examined whether the protective mechanism of curcumin is HO-dependent using a specific HO-activity inhibitor. We used the synthetic isozyme-selective non-porphyrin HO-activity inhibitor QC-15 to test its effect on ASC survival. QC-15 is an imidazole-dioxolane compound that binds to the distal side of the heme-binding place in the HO protein and is a highly selective HO-1 activity inhibitor and has less potency to inhibit HO-2 activity [74,75,76,77].

Specifically, we investigated whether HO-1 activity inhibition would reduce ASC survival, and in addition, if the protecting effect of curcumin could be attenuated by co-treatment with QC-15. ASCs were therefore pre-incubated for 24 h with HO-activity modulators followed by a co-treatment with 350 µM H_2_O_2_ for 24 h, after which the ASC-survival was assessed using the Alamar blue assay.

**Figure 5:**
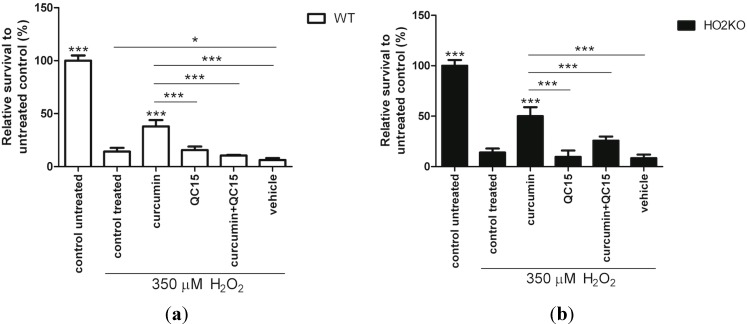
Inhibition of HO-activity abrogated the protective effect of curcumin against H_2_O_2_-induced cell death in isolated WT and HO-2 KO ASCs. (**a**) WT ASC and (**b**) HO-2 KO ASC survival after 24 h pre-treatment with HO-modulators or untreated (control) and 24 h co-treatment together with 350 µM H_2_O_2_ as measured by Alamar blue assay and fluorimetric quantification, related to untreated control. Vehicle consisted of 0.5% ethanol (curcumin solvent) in culture media. * is significant different from treated control. (* *p* < 0.05, and *** *p* < 0.001). Data represent a representative of three independent experiments and present mean ± SD. For each condition six samples were analyzed.

Surprisingly, we found that co-exposure of 50 µM QC15 together with H_2_O_2_ did not influence cell survival when compared to cells treated with only H_2_O_2_ in both WT and HO-2 KO ASCs (Figure 5). This suggests that the basal levels of both HO-1 and HO-2 are not sufficient for protection. However, the increased survival of ASCs after 10 µM curcumin treatment, and subsequent induced HO-1 expression, against H_2_O_2_ was completely abrogated (*p* < 0.001) following co-treatment with 50 µM QC15 in both WT as HO-2 KO ASCs. These results clearly demonstrate the protective potential of pre-induction of HO-1-activity in preventing oxidative stress-induced ASC death. Surprisingly, this is likely independent from HO-2, since no differences in survival were found between WT and HO-2 KO ASCs. In addition, picogreen data also showed a decline in cell amount after co-treating curcumin with QC15 (Figure S2). However, this decrease in cell amount was not always significant for both WT and HO-2 KO ASCs in replicate experiments. Similar protective effects of HO-activity from injurious insults have also been demonstrated in other cell lines [78,79]. Induction of HO-activity by flavonoid baicalcin and HO-1 transfection protected raw 264.7 macrophages and vascular smooth muscle cells against H_2_O_2_-induced cell death. Also here, inhibition of HO-activity attenuated this protection [78,79].

Thus, curcumin-induced protection against H_2_O_2_-induced ASC death acts via induction of HO-1, whereas inhibition of HO-activity abrogates this protective effect.

### 2.6. Do Anti-Oxidants Bilirubin, Biliverdin, and NAC Rescue from H_2_O_2_-Induced ASC Death?

**Figure 6:**
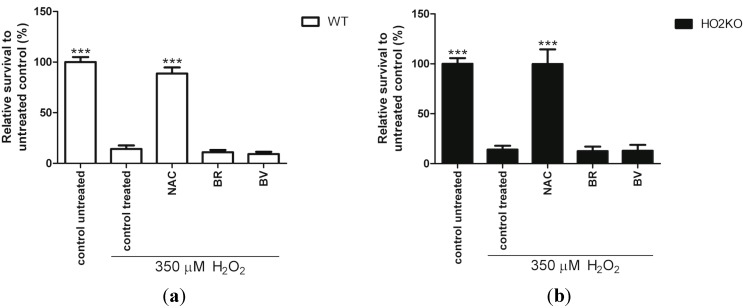
Survival of WT and HO-2 KO ASC against H_2_O_2_-induced cell death after treatment with anti-oxidants. (**a**) WT ASC and (**b**) HO-2 KO ASC survival after 24 h pre-treatment with anti-oxidant NAC or HO-effector molecules BR and BV, and 24 h co-treatment together with 350 µM H_2_O_2_ following an Alamar blue assay and fluorimetric quantification, related to untreated control. * is significant different from treated control. (*** *p* < 0.001). Data represent a representative of three independent experiments and present mean ± SD. For each condition six samples were analyzed. NAC: *N*-acetylcysteine; BR: Bilirubin; BV: Biliverdin.

Since HO-activity protects against H_2_O_2_-mediated cell death, we investigated whether the anti-oxidative properties of HO-effector molecules BR and BV, or anti-oxidant *N*-actylcysteine (NAC) could mediate a protective effect. It has previously been reported that NAC; a precursor of glutathione (GSH) synthesis, and BR and BV can protect cells from ROS-induced cell death [62,80,81]. GSH serves as electron donor for glutathione peroxidase to catalyze the reduction of H_2_O_2_ into H_2_O, and is together with catalase the only defense available to metabolize H_2_O_2_ [82,83]. GSH and BR are both prominent endogenous anti-oxidant molecules, protecting against oxidative stress on a complementary basis and have a distinct antioxidant mechanism [84]. Therefore, we investigated the possible contribution of the different anti-oxidants NAC, BR, and BV on ASC survival. Pre-treatment for 24 h with 10 µM BR, 10 µM BV, and 6 mM NAC and co-exposure for another 24 h with 350 µM H_2_O_2_ was performed to investigate the effects on ASC survival. This was assessed using the Alamar blue assay. Anti-oxidant NAC significantly increased cell survival compared to H_2_O_2_-treated control in WT and HO-2 KO ASCs 6.3- and 7.1-fold, respectively (Figure 6). NAC may also influence mitochondrial biogenesis [85], and thus might cause bias in the Alamar blue assay, since this assay is a measure of mitochondrial activity. However, NAC significantly increased the cell amount compared to H_2_O_2_-treated control as confirmed by the picogreen assay, suggesting that NAC does not only promote mitochondrial biogenesis but also cell survival (Figure S2). Since NAC can interfere with several cell viability test methods [86], we corroborated that cell-free NAC containing media did not influence the Alamar blue signal (data not shown). Increasing GSH formation results in a more reduced intracellular environment, and promotes cell proliferation and survival [87]. NAC has been demonstrated to promote the proliferation of different cell types, including adipose derived stem cells and BM stromal cells [88,89]. Surprisingly, the anti-oxidants BR and BV did not improve ASC survival in both WT and HO-2 KO ASCs (Figure 6 and S2). Treatment with BR and BV in the absence of H_2_O_2_ did not result in increased cell death, as measured by the Alamar blue assay (data not shown). The observed differences between NAC and BR/BV may be related to the different targets that these anti-oxidants have [84]. GSH is hydrophilic and protects mainly water-soluble proteins in the cytosol, while BR and BV are more lipophilic and protect against lipid peroxidation of cell membranes [84]. The microenvironment largely determines which mechanism is needed to sustain redox homeostasis [87]. H_2_O_2_ can easily penetrate the cell membrane, and form hydroxyl radicals with intracellular metal ions. Treatment with H_2_O_2_ results in cytosolic release of cytochrome c as well as activating caspase-9 and caspase-3 and works through the intrinsic/mitochondrial apoptotic pathway in several cell types, including MSCs [90,91,92,93,94,95]. And hence, H_2_O_2_ scavenging is likely more dependent on GSH, rather than BR and BV.

Our results show that H_2_O_2_-induced cytotoxicity can be prevented by NAC, but not BR and BV. This suggests that HO-1-mediated ASC survival following H_2_O_2_-treatment is independent from HO-effector molecules BR and BV and independent of intrinsic HO-2 expression.

### 2.7. Does HO-Effector Molecule Carbon Monoxide Influence H_2_O_2_-Induced ASC Death?

Since we have shown that HO-activity is responsible for ASC survival, and is independent from the effector molecules BR and BV, we next investigated the effects of the HO-effector molecule CO. The HO-effector molecules also provide protection through different mechanisms; BR and BV are anti-oxidants, whereas CO is thought to work on regulating downstream signaling pathways. Furthermore, CO may produce mitochondrial ROS initiating intracellular protective signaling pathways and maintain cells in homeostasis [96,97,98].

**Figure 7:**
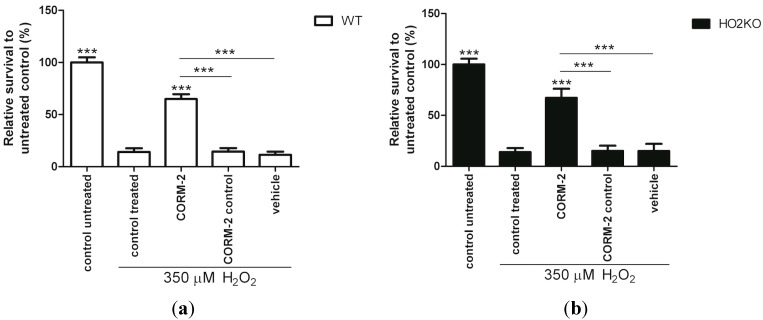
CORM-2 increases WT and HO-2 KO ASC survival against H_2_O_2_-induced cell death. (**a**) WT ASC and (**b**) HO-2 KO ASC survival after 24 h pre-treatment with CORM-2, or its controls, and 24 h co-treatment together with 350 µM H_2_O_2_ following an Alamar blue assay and fluorimetric quantification, related to untreated control. Vehicle consisted of 0.1% DMSO (CORM-2 and CORM-2 control solvent) in culture media. * is significant different from treated control. (*** *p* < 0.001). Experiments are performed in triplicate with samples in sextet. Representative graph is shown. Data are presented as mean ± SD. CORM-2: CO-releasing molecule-2.

In order to determine whether CO could mediate the HO-induced protection against H_2_O_2_-induced ASC death, we treated the cells with CO releasing molecules-2 (CORM-2), and found that this resulted in significantly higher ASC survival in WT and HO-2 KO ASCs compared to H_2_O_2_-exposed ASCs (Figure 7). The absence of HO-2 expression in the ASCs in our experimental setup did not have any significant influence on its survival against H_2_O_2_-induced cell death. In addition, CORM-2 control and solvent control (DMSO) treated cells showed no protection and survival were comparable to cells treated with H_2_O_2_. Thus, the HO-mediated protection mechanism is likely dependent on the effects of CO-release. This was partly confirmed by our picogreen data (Figure S2). Here, we found that more cells were present after CORM-2 treatment in WT ASCs (*p* < 0.001) exposed to H_2_O_2_. However, in HO-2 KO ASCs this protective effect of CORM-2 on cell amount was not always significant in replicate experiments. A decrease in viability may therefore not always mean a decrease in cell amount. In line with our results, Lin *et al.* [78] also demonstrated that CO, but not BR and BV administration inhibited H_2_O_2_-induced cytotoxicity in macrophages, suggesting that CO is responsible for the protective effect caused by HO-1 overexpression in both macrophages and ASCs. Furthermore, the ruthenium-based CORM-2 and CORM-2 control have been demonstrated to be potent inducers of HO-1, which could have further boosted the protective effects of CORM-2 via a positive feedback loop [99,100]. Preliminary experiments show indeed induction of HO-1 following exposure to CORM-2 and to a lower extent for CORM-2 control. This positive feedback between HO-1 and CO (and nrf-2) has previously been demonstrated in hepatocytes [101] and vascular endothelium cells [102].

The down-stream protective effects of CO can be mediated by several protective signaling pathways. Pre-treatment with CORM-2 increases mitochondrial ROS signaling, which leads to pre-conditioning of the cells, making them more resistant to secondary stresses with H_2_O_2_ [103,104]. In addition, CO may activate diverse other downstream signaling pathways, including nrf-2, guanylate cyclase, p38 mitogen-activated protein kinase (MAPK), PI3K-Akt, and iNOS [98,102,104]. Finally, CO can reduce the production of ROS in cells treated with H_2_O_2_, probably by preventing the formation of hydroxyl radicals by binding to ionized iron [105].

More research is warranted to unravel the exact mechanism by which CORM-2 exerts its protective effects. These results suggest that CO is responsible for the observed HO-mediated ASC survival from oxidative stress.

### 2.8. In Summary

Administrating MSCs forms a promising therapy following pathologic tissue injury. However, the poor survival of MSCs following administration limits their therapeutic efficacy. In this study, we demonstrated that exposure of adipose-derived MSCs (ASCs) to oxidative stress results in ASC death and that induction of the HO-system by curcumin can attenuate this. Simultaneous inhibition of HO-activity could abrogate the protective effects of curcumin, demonstrating an essential role for HO-1. We found that also NAC improved ASC survival, whereas BR or BV did not demonstrate a protective effect, and thus suggest differential anti-oxidative protective mechanisms. Since administration of the HO-effector molecule CO also attenuated H_2_O_2_-induced ASC death, it is likely that CO mediates in part the protective effects of HO-1 induction. All these effects were independent from HO-2 expression, as the results for WT and HO-2 KO ASCs were similar.

### 2.9. Clinical Relevance

Increased levels of ASCs at the site of injury may ameliorate tissue repair by their differentiation capacity and the excretion of paracrine factors into the tissue environment further promoting wound repair [106,107,108,109]. The clinical relevance of HO-1 induction in MSCs is further supported using *in vivo* models of tissue injury demonstrating improved MSC survival and tissue function [33,34,35,109,110,111,112].

Our results further emphasize the important roles of HO-1 and CO as targets for improved ASC survival and therapy in tissue repair. We also show that HO-2 expression is less important in mediating protection against oxidative insults in ASCs.

## 3. Experimental Section

### 3.1. Reagents

Biliverdin and bilirubin were purchased from Frontier Scientific, Carnforth, UK. Curcumin, *N*-acetyl-l-cysteine (NAC), tricarbonyldichlororuthenium(II)dimer (CO releasing molecule: CORM-2), and Ruthenium(III)chloride (CORM-2 control) were purchased from Sigma-Aldrich, Zwijndrecht, The Netherlands. Biliverdin, and bilirubin were prepared by dissolving this together with Trizma base in 2 mL 0.1 M NaOH and 5 mL H_2_O. The pH was adjusted to pH 8 with HCl, and further diluted till 10 mL with H_2_O. An end concentration of 1 µM was used in the experiments [76]. Curcumin was dissolved in 99.5% ethanol at a concentration of 2 mM. The stock solutions were filter-sterilized (0.2 μm filter), protected from light and directly used at a final concentration of 5–15 µM. NAC was dissolved in phosphate buffered saline (PBS) and used at a final concentration of 6 mM [76]. CORM-2, and CORM-2 control were both dissolved in DMSO, and diluted 1000× in work solution to 50 μM. QC15 was produced by Dr. Szarek; Department of Chemistry, Queen’s University, Kingston, ON, Canada [62,113], and used at an end concentration of 50 µM. Hydrogen peroxide (H_2_O_2_) (30%) was purchased from Merck, Hohenbrunn, Germany.

### 3.2. Mice

The Committee for Animal Experiments of the Radboud University Nijmegen approved all procedures involving animals. Mice (HO-2 KO and WT with a mixed C57BL/6_129/Sv background [114]) of 6–12 weeks in age were housed under standard specific pathogen-free housing conditions.

### 3.3. Isolation and Culture of ASCs

Adipose-derived mesenchymal stem cells (ASCs) were routinely isolated from WT and HO-2 KO mice as described previously [52]. In brief, adipose tissue surrounding the mouse inguinal lymph nodes were isolated and cut up using scalpels, followed by 30 min of incubation at 37 °C with digestion buffer (30 wt/v %, fat/digestion buffer). Digestion buffer consisted of complete ASC culture media: DMEM/F12 (Gibco, New York, NY, USA) supplemented with 2% Penicillin/Streptomycin (Pen/Strep) (Invitrogen, Carlsbad, CA, USA), 0.5% Amphotericin B (Invitrogen), 16 μM Biotin (Sigma Aldrich, St. Louis, MO, USA), 18 μM Panthotenic Acid (Sigma Aldrich), 100 μM Ascorbic Acid (Sigma Aldrich), and 10% Newborn Calf Serum (Sigma Aldrich), with additionally 2 mg/mL collagenase A (Roche, Woerden, The Netherlands) and 2 wt/v % Bovine Serum Albumin (Sigma Aldrich). Digested adipose tissue was filtered over a 25 μm filter and the filtrate was centrifuged 10 min at 500× *g*. Pellet was resuspended in 1 mL culture media and red blood cells were lysed with 7 mL ammonium chloride solution (Stem Cell technologies, Grenoble, France). Next, the cells were counted using the Beckman Coulter Z2 (Beckman Coulter, Woerden, The Netherlands: >4 μm <25 μm) and seeded at a concentration of 8000 cells/cm^2^ in complete culture media.

Genotypes were confirmed using qPCR showing that HO-2 KO ASCs were in contrast to WT ASCs, negative for HO-2 mRNA expression (data not shown). In order to phenotypically analyze the ASC population we further used a panel of specific ASC markers and exclusion markers expressed on macrophages, endothelial cells, and hematopoietic cells to exclude non-ASCs. Cells (passage 3) isolated from WT and HO-2 KO mice were tested for six different stem cell markers using flow cytometry. The adipose-derived cells were stained with the MSC markers Sca-1, CD44 and CD105 (BD Bioscience, Breda, The Netherlands; Biolegend, San Diego, CA, USA and eBioscience, San Diego, CA, USA, respectively) and exclusion markers CD11b, cKit and CD34 (Biolegend; BD Bioscience and eBioscience, respectively). In addition, we chose a subset of cell markers for Quantitative Reverse-Transcriptase PCR (qPCR), analysis (Table 1). ASCs up to passage number 5 were used for the different experiments.

**Table 1:** Primer sequences of mouse ASC cell surface markers and cytoprotective enzymes.

Gene Name	Sense	Antisense
*Cell Surface Marker*		
*CD45*	5'-GACAGAGTGCAAAGGAGACC-3'	5'-ATCACTGGGTGTAGGTGTTTG-3'
*Sca1*	5'-AGCAGTTATTGTGGATTCTC-3'	5'-TAGTACCCAGGATCTCCATAC-3'
*CD105*	5'-TTGTACCCACAACAGGTCTC-3'	5'-GGTGGTAAACGTCACCTCAC-3'
*CD29*	5'-AAATTGAGATCAGGAGAACCAC-3'	5'-GGTAATCTTCAGCCCTCTTG-3'
*CD11b*	5'-CTGGTCACAGCCCTAGCC-3'	5'-TTTGCATTCTCTTGGAAGGTC-3'
*CD31*	5'-CTGGTGCTCTATGCAAGC-3'	5'-GCTGTTGATGGTGAAGGAG-3'
*CD34*	5'-TGAGTCTGCTGCATCTAAATAAC-3'	5'-CTCATTGGTAGGAACTGATGG-3'
*CD117*	5'-GCCAGACAGCCACGTCTC-3'	5'-CTGATTGTGCTGGATGGATG-3'
*CD106*	5'-CGTGGACATCTACTCTTTCC-3'	5'-TGTAAACTGGGTAAATGTCTGG-3'
*CD86*	5'-GTCAGTGATCGCCAACTTC-3'	5'-TCTTCTTAGGTTTCGGGTGAC-3'
*Housekeeping Gene*		
*GAPDH*	5'-GGCAAATTCAACGGCACA-3'	5'-GTTAGTGGGGTCTCGCTCCTG-3'
*Cytoprotective Enzymes*		
*HO-1*	5'-CAACATTGAGCTGTTTGAGG-3'	5'-TGGTCTTTGTGTTCCTCTGTC-3'
*HO-2*	5'-AAGGAAGGGACCAAGGAAG-3'	5'-AGTGGTGGCCAGCTTAAATAG-3'

### 3.4. Heme Oxygenase Protein Expression by in-Cell Western

ASCs were cultured in a 96-well plate, fixated with 4% paraformaldehyde (PFA) for 15 min, and permeabilized with 0.1% Triton X-100 in PBS for 30 min. The cells were blocked with 5% ELK (Campina skimmed milk powder) in PBS for 90 min. First antibody (rabbit-anti-HO-1 polyclonal antibody; Stressgen Biotechnologies, Victoria, BC, Canada; cat# SPA895: 6.7 μg/mL in 2.5% ELK and mouse-anti-β-actin monoclonal antibody; Sigma Aldrich, St. Louis, MO, USA; cat# A5441: 5 μg/mL in 2.5% ELK) treatment was performed overnight at 4 °C. Secondary antibody treatment (goat-anti rabbit Alexa fluor 680; Invitrogen: Molecular Probes, Eugene, OR, USA, 2.5 μg/mL in 2.5% ELK, and goat-anti-mouse InfraRedDye 800; Rockland, Gilbertsville, PA, USA, 1.25 μg/mL in 2.5% ELK) was performed for one hour. The plate was measured using the Odyssey Imager with detection in both 700 and 800 nm channels at an intensity of 5 and 7.5, respectively, and was analyzed using Odyssey software (LI-COR Biosciences, Lincoln, NE, USA; version 2.1.12). The amount of HO-1 protein expression was quantified in relation to the expression of β-actin protein. Since the Western blot shows only one specific single band following induction with curcumin, the SPA895 antibody specifically recognizes HO-1 (see Figure S3). Therefore, we used this antibody for immuno-fluorescent and In-Cell Western techniques.

### 3.5. Heme Oxygenase Protein Expression by Immuno-Fluorescent Staining

HO-1 protein expression was also evaluated by immuno-fluorescent staining of ASCs treated with curcumin on plastic chamberslides (Nunc, Lab-Tek: Permanox 8-well chamberslides: Thermo Scientific, Rochester, NY, USA). In the chambers, cells were fixated with 4% PFA for 15 min, permeabilized with 0.5% Triton X-100 in PBS for 20 min, and washed with 0.05% Tween in PBS (PBS-T). Next, the cells were incubated with blocking buffer consisting of 2% bovine serum albumin, 2% normal goat serum, 0.1% Triton X-100, 0.05% Tween, and 100 mM glycin in PBS for 30 min. First antibody (Rb-a-HO-1; Stressgen Biotechnologies; Cat# SPA895) was diluted 1:600 in blocking buffer without glycine and incubated on cells for 60 min. After washing with PBS-T, cells were incubated with secondary antibody goat-anti-rabbit alexa 594 (Life Technologies, Bleiswijk, The Netherlands, cat# A-11037 diluted 1:200 in blocking buffer without glycin) for 60 min. Next, the cells were washed with PBS-T, followed by washing with PBS, and nuclei were stained with 4',6-diamidino-2-phenylindole (DAPI) for 10 min. Finally, cells were washed with PBS, 1:1 PBS:MQ, and MQ and sealed with 1,4-diazabicyclo[2.2.2]octaan (DABCO). The Zeiss Imager Z1 microscope (Zeiss, Sliedrecht, The Netherlands) was used to make fluorescent pictures of the cells using Axiovision software version 4.8 (Zeiss).

### 3.6. mRNA Isolation and Quantitative-Reverse-Transcriptase-PCR

Cells were lysed and homogenized in TRIzol (Invitrogen) and RNA was further extracted with the RNeasy mini kit (Qiagen, Venlo, The Netherlands). DNase treatment (Qiagen: RNase-Free DNase Set) was performed between the first washing steps with RW1 buffer. RNA concentration was determined using the nanodrop 2000 spectrophotometer (Thermo Scientific). For the reverse transcriptase treatment 1 µg sample RNA together with total RT mix (iScript cDNA Synthesis kit from Bio-Rad Laboratories, Veenendaal, The Netherlands) in a total volume of 20 µL was incubated for 5 min at 25 °C, 30 min at 42 °C and 5 min at 85 °C. Hereafter the cDNA was diluted 10× and used for Quantitative Reverse-Transcriptase PCR (qPCR), using CFX96 Real-Time System (Bio-Rad Laboratories). The reaction was performed in 25 μL containing 5 μL cDNA, 0.6 μM primers, 12.5 μL iQ SYBR Green Supermix (Bio-Rad Laboratories). After incubation of 3 min, amplification was carried out for 40 cycles of 15 s at 95 °C and 30 s at 60 °C. The melting temperature of the products was defined to indicate amplification specificity. All values were normalized to the housekeeping gene *GAPDH*. Data of the ASC characterization were presented as 2^−Δ*C*t^. HO-1 mRNA expression was evaluated after treatment with curcumin for 24 h and related to untreated control (2^−ΔΔ*C*t^). All used primers are summarized in Table 1.

### 3.7. Determination of Cell Viability

Cells were seeded into a 96 wells plate at 2000 cells/well and after overnight culturing of the cells were pre-treated with HO-activity modulators or HO-system related molecules for 24 h, followed by a co-treatment with 350 µM hydrogen peroxide (selected from a 100–500 µM H_2_O_2_ dose range) for 24 h in 100 μL media. Hereafter, media was removed and replaced by media containing 10% AlamarBlue^®^ Cell Viability Reagent (Invitrogen) and further incubated at 37 °C for three hours. The oxidized form of this Alamar blue (resazurin) enters the cytosol where it is converted into the reduced form (resorufin) by mitochondrial enzyme activity. The amount of resorufin is directly proportional to the amount of proliferating viable cells and was measured fluorescently at 530/590 nm using the Universal Microplate reader FL600 (Bio-TEK instruments Inc., Winooski, VT, USA). Viability was calculated as percent of the difference in reduction of Alamar blue in treated *versus* non-treated cells, corrected for background signal of Alamar blue: Viability = (Ex-Eblanc)/(Ec-Eblanc) × 100; where Ex = extinction treatment, Ec = Extinction untreated control, and Eblanc = extinction Alamar blue background.

### 3.8. Determination of Cell Amount

The influence of pre- and co-treatment with HO-modulators and effector molecules on cell loss after H_2_O_2_-treatment was determined using PicoGreen dsDNA Quantification reagent (Molecular Probes Inc., Eugene, OR, USA). The amount of double stranded DNA can be quantified using this assay and this correlates to the amount of cells. After measuring the Alamar plate, the media was removed and the plate was washed twice with PBS. Cells were lysated by adding 0.1% Triton X-100 and frozen (−80 °C) and thawed (37 °C) for three consecutive times. The assay was performed according to the protocol of the manufacturer. The fluorescent signal was measured in a FL600 Microplate Fluorescence Reader (Bio-Tek Instruments Inc., Winooski, VT, USA) at excitation 485 nm, emission 520 nm. DNA standards ranging from 0 to 2000 ng DNA/mL were used. The total amount of DNA after treatment was calculated using the standard curve.

### 3.9. Statistical Analysis

Data were analyzed using GraphPad Prism 5.01 software (San Diego, CA, USA). Outliers were tested using the Grubbs’ test. In Alamar blue experiments, one outlier was detected in a control untreated group of WT ASCs in two experiments. In picogreen experiments, one outlier was found in a control treated group of WT ASCs and one outlier was found in NAC treated group of HO-2 KO ASCs, in single experiments. Data was analyzed by one- or two-way analysis of variance (ANOVA) with Bonferroni’s multiple comparison post test. One-way ANOVA was used in survival studies and picogreen studies and two-way ANOVA was used in other experiments. Results were considered significantly different when *p* < 0.05 (* *p* < 0.05, ** *p* < 0.01, and *** *p* < 0.001).

## 4. Conclusions

Survival from H_2_O_2_-induced apoptosis was similar for WT and HO-2 KO ASCs, suggesting that the HO-2 isoform is not able to provide protection in this specific setting. ASC survival was ameliorated following pre-induction of HO-1 using the cytoprotective flavonoid curcumin. This protection by curcumin was mediated by HO-1 activity since simultaneous inhibition of HO-activity abrogated this curcumin-mediated protection. HO-effector molecules BR/BV did not provide protection, whereas CO protected against H_2_O_2_-induced cell death, suggesting involvement of CO in HO-1 mediated protection. HO-1 pre-induction by curcumin or CO exposure could therefore form a novel promising adjuvant strategy to promote stem cell survival during therapy.

## Supplementary Information

Supplementary materials can be found at http://www.mdpi.com/1422-0067/15/10/17974/s1.

## Supplementary Files

Supplementary File 1
