# Supplementary material for: Curcumin-Induced Heme Oxygenase-1 Expression Prevents H2O2-Induced Cell Death in Wild Type and Heme Oxygenase-2 Knockout Adipose-Derived Mesenchymal Stem Cells
Source: Int J Mol Sci. 2014 Oct 8;15(10):17974–99. doi: 10.3390/ijms151017974 (PMC4227200; doi:10.3390/ijms151017974)
Supplement: Supplementary File 1 [file ijms-15-17974-s001.pdf]

Supplementary Information

Supplemental Experimental Section

Heme Oxygenase-1 Protein Expression by Western Blot

ASCs were cultured in a 6-well plate in the presence of 0, 5, and 10 μM of curcumin for 24 h. After treatment, cells were harvested using trypsin-EDTA, pelleted by centrifugation and protein was extracted by resuspending the cell pellet for 30 min on ice in lysis buffer consisting of 1 mM EDTA, 0.5% triton-X-100, 100 μM phenylmethylsulfonyl fluoride (PMSF) in ethanol, and 1:100 complete mini protease inhibitor (Roche). Cell lysates were centrifuged for 2 min at 13,000 rpm at 4 °C and 7.5 μg total protein was separated by SDS/PAGE using a 10% gel. Subsequently, proteins were blotted onto a nitrocellulose membrane using the iBlot system (Invitrogen). The membrane was blocked for 30 min in 5% ELK in PBS and overnight incubated with first antibodies (rabbit-anti-HO-1 polyclonal antibody; Stressgen Biotechnologies, Victoria, BC, Canada; 1:5000 and mouse-anti-β-actin monoclonal antibody; Sigma-Aldrich, St. Louis, MO, USA, 1:100,000) in 2.5% ELK containing 0.1% Tween-20. Afterwards, the membrane was briefly washed three times with PBS and twice with PBS containing 0.1% Tween-20 for 10 min. The secondary antibodies (goat-anti rabbit Alexa fluor 680; Invitrogen: Molecular Probes, Eugene, OR, USA, 1:10,000, and goat-anti-mouse InfraRedDye 800; Rockland, Gilbertsville, PA, USA, 1:10,000) dissolved in 2.5% ELK containing 0.1% Tween-20 and 0.01% SDS were incubated for 45 min at room temperature. After thorough washing, the membrane was measured using the Odyssey Imager with detection in both 700 and 800 nm channels at an intensity of 3.5 and 7.5, respectively. The intensity of the bands of the Western blot was analyzed using Odyssey software (LI-COR Biosciences; version 2.1.12). The amount of HO-1 protein expression was quantified in relation to the expression of β-actin protein.

Detection of Apoptosis by Flow Cytometry

Flow cytometry was used to study the effects of H_2O_2 on apoptosis, by double staining with fluorescein isothiocyanate (FITC) labeled—Annexin V and propidium iodide (PI). In short, untreated and H_2O_2-treated ASCs were resuspended in binding buffer and stained with Annexin V-FITC and PI for 15 min at RT in the dark, according to manufacturer's protocol (BioVision Inc., ITK Diagnostics, Uithoorn, The Netherlands). Cells treated with 4% paraformaldehyde containing 0.1% saponine and stained with Annexin V and PI were used as positive control. Detection of Annexin V-FITC and PI binding was performed by a FACSCalibur (Becton Dickinson Biosciences, San Jose, CA, USA) using channels FL-1 (Annexin V-FITC) and FL-3 (PI). Viable cells (Annexin V^-/PI^-), early apoptotic cells (Annexin V^+/PI^-), late apoptotic cells (Annexin V^+/PI^+), and necrotic cells (Annexin V^-/PI^+) were quantified as a percentage of the gated population, using FlowJO software (version 7.6.5 for windows, Tree Star, Inc., Ashland, OR, USA).

Figure S1. Analysis of apoptosis of WT and HO-2 KO ASCs after treatment with 350 µM H_2O_2 or control for 24 h using Annexin V-FITC (FL-1) and propidium iodide (PI) (FL-3) labeling. It was found that both WT and HO-2 KO ASCs showed similar induction of apoptosis following H_2O_2 treatment.

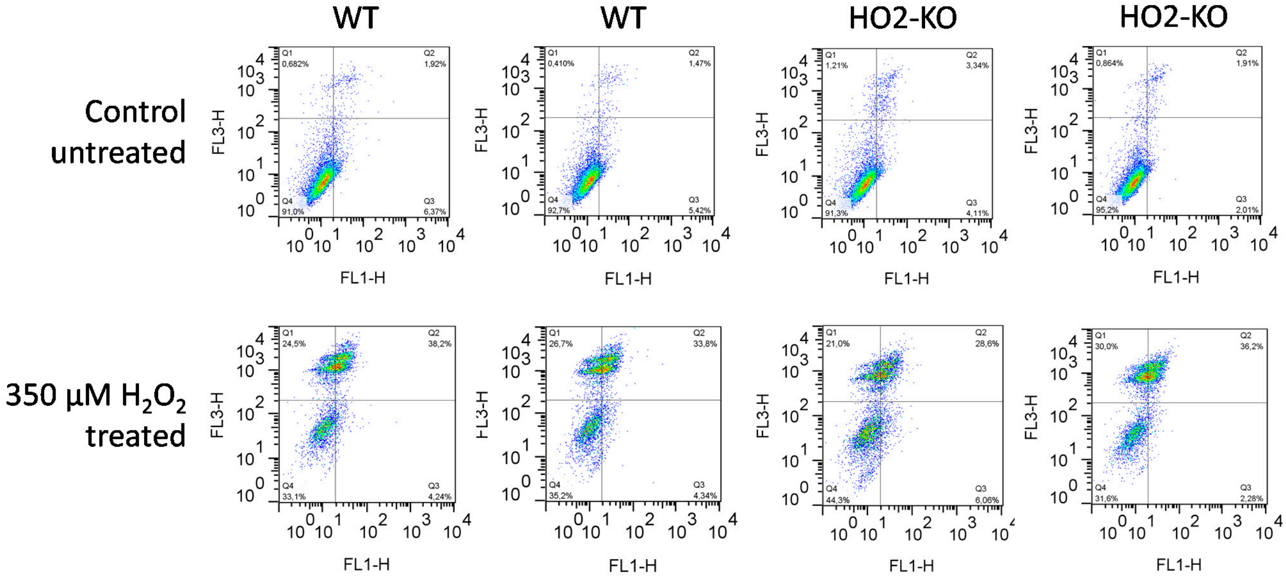

Figure S2. Number of WT and HO-2 KO ASCs after treatment with HO-system related molecules and H_2O_2-induced cell death. (**a**) WT ASC and (**b**) HO-2 KO ASC after 24 h pre-treatment with several agents, and 24 h co-treatment together with 350 µM H_2O_2 following a picogreen assay and fluorimetric quantification, related to untreated control. DMSO control represents the vehicle for CORM-2 and CORM-2 control and consisted of 0.1% DMSO in culture media. Ethanol control is the vehicle for curcumin and consisted of 0.5% ethanol in culture media. * is significant different from treated control (** $p < 0.01$, and *** $p < 0.001$). Experiments are performed in triplicate with samples in sextet. Representative graph is shown. Data are presented as mean ± SD. NAC: *N*-acetylcysteine; CORM-2: CO-releasing molecule-2; BR: Bilirubin; BV: Biliverdin.

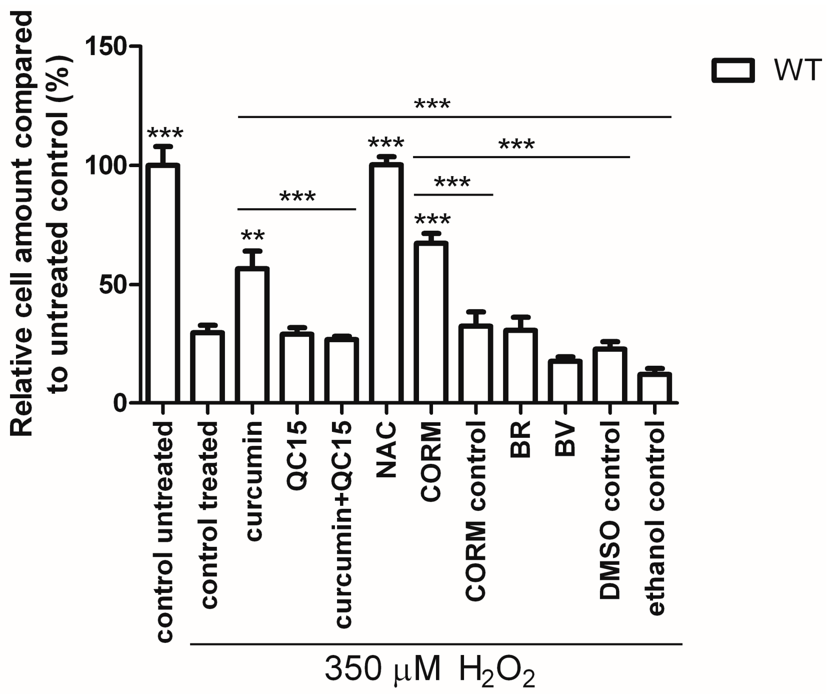

(**a**)

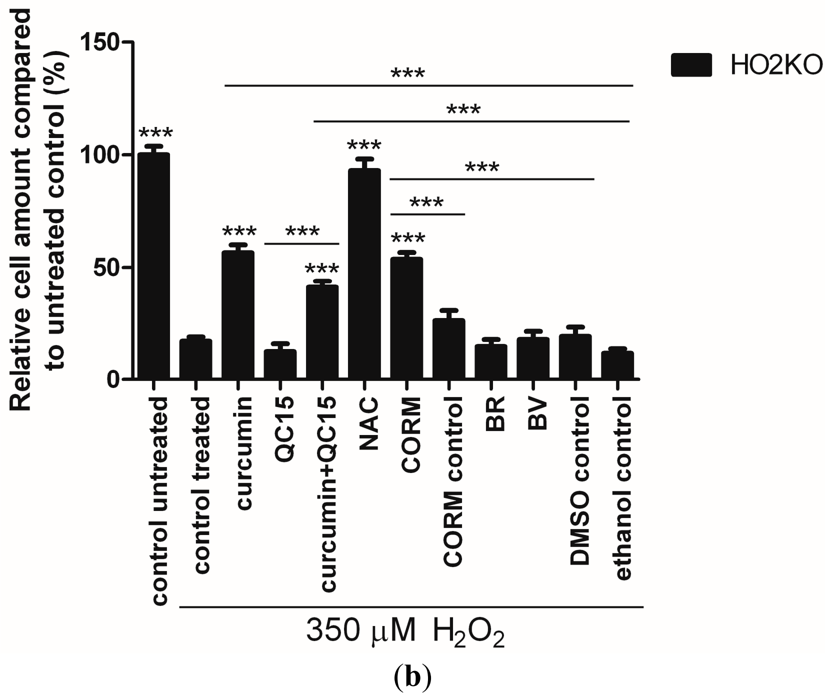

(**b**)

Figure S3. HO-1 protein expression was induced with curcumin after 24 h treatment in WT and HO-2 KO ASCs. (**a**) WT and HO-2 KO ASCs were treated with curcumin (0–10 μM) for 24 h and HO-1 protein expression was analyzed using Western blot, and housekeeping protein β-actin was used to correct for the amount of cells. Experiment was performed once; (**b**) Quantification of the HO-1 protein expression of the Western blot, corrected for the amount of cells with β-actin and related to untreated control.

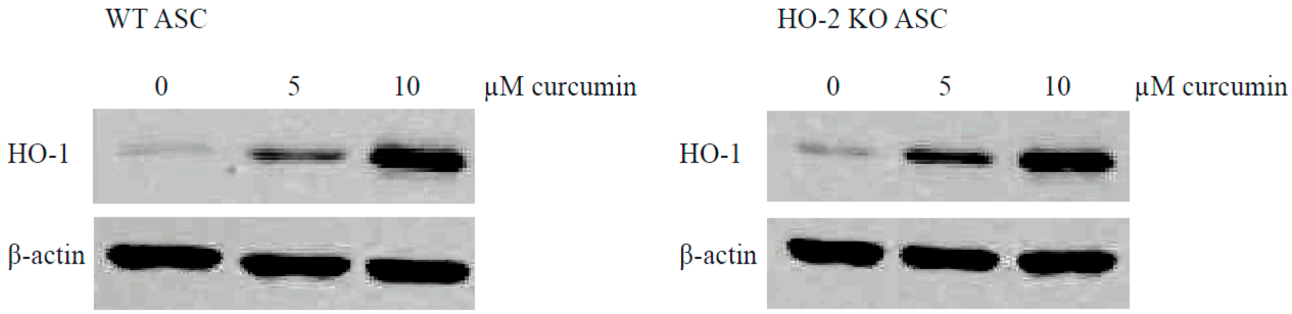

(**a**)

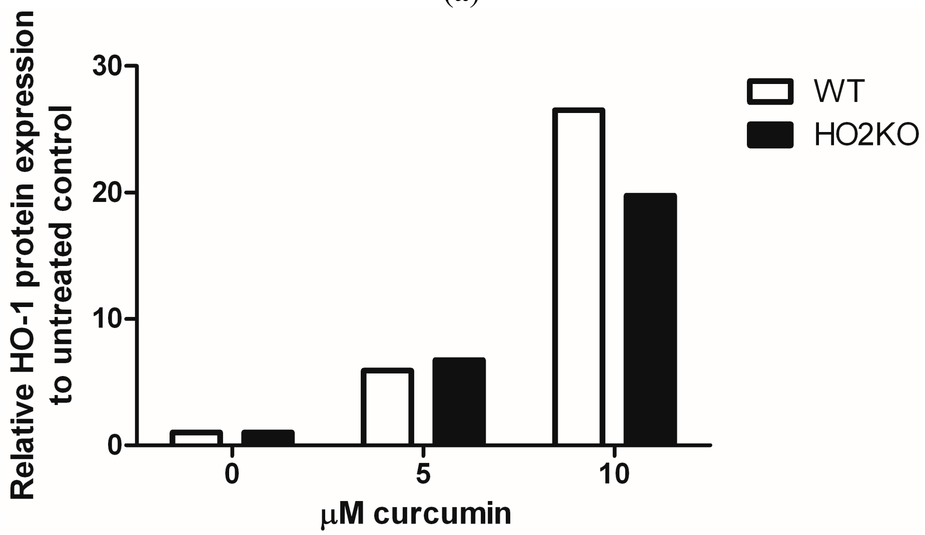

(**b**)
